# Genetic polymorphism of histidine rich protein 2 in *Plasmodium falciparum* isolates from different infection sources in Yunnan Province, China

**DOI:** 10.1186/s12936-019-3084-4

**Published:** 2019-12-30

**Authors:** Ying Dong, Shuping Liu, Yan Deng, Yanchun Xu, Mengni Chen, Yan Liu, Jingpo Xue

**Affiliations:** 1 Yunnan Institute of Parasitic Diseases Control, Yunnan Provincial Key Laboratory, Yunnan Centre of Malaria Research, Academician Workstation of Professor Jin Ningyi, Pu’er, 665000 China; 2 School of Basic Medical Sciences, Dali University, Dali, 667000 China; 3 National Institute of Parasitic Diseases, Chinese Center for Disease Control and Prevention, Shanghai, 200025 China

**Keywords:** Yunnan, Different infection sources, *Plasmodium falciparum*, Histidine-rich protein 2, Peptid chain, Deletion polymorphism

## Abstract

**Background:**

Failed diagnoses of some falciparum malaria cases by RDTs are constantly reported in recent years. *Plasmodium falciparum* histidine-rich protein 2 (*pfhpr2)* gene deficiency has been found to be the major reason of RDTs failure in many countries. This article analysed the deletion of *pfhpr2* gene of falciparum malaria cases isolated in Yunnan Province, China.

**Methods:**

Blood samples from falciparum malaria cases diagnosed in Yunnan Province were collected. *Plasmodium* genomic DNA was extracted and the *pfhrp2* gene exon2 region was amplified via nested PCR. The haplotype of the DNA sequence, the nucleic acid diversity index (PI) and expected heterozygosity (He) were analyzed. Count *Pf*HRP2 amino acid peptide sequence repeat and its times, and predict the properties of *Pf*HRP2 peptide chain reaction to RDTs testing.

**Results:**

A total of 306 blood samples were collected, 84.9% (259/306) from which *pfhrp2* PCR amplification products (gene exon2) were obtained, while the remaining 47 samples were false amplification. The length of the 250 DNA sequences ranged from 345 - 927 bp, with 151 haplotypes, with PI and He values of 0.169 and 0.983, respectively. The length of the *Pf*HRP2 peptide chain translated from 250 DNA sequences ranged from 115 to 309 aa. All peptide chains had more than an amino acid codon deletion. All 250 *Pf*HRP2 strands ended with a type 12 amino acid repeat, 98.0% (245/250) started with a type 1 repetition and 2.0% (5/250) with a type 2 repetition. The detection rate for type 2 duplicates was 100% (250/250). Prediction of RDT sensitivity of *Pf*HRP2 peptide chains based on type 2 and type 7 repeats showed that 9.60% (24/250), 50.0% (125/250), 13.20% (33/250) and 27.20.5% (68/250) of the 250 peptide chains were very sensitive, sensitive, borderline and non-sensitive, respectively.

**Conclusion:**

The diversified polymorphism of the *pfhrp2* gene deletion from different infection sources in the Yunnan province are extremely complex. The cause of the failure of *pfhrp2* exon2 amplification is still to be investigated. The results of this study appeal to Yunnan Province for a timely evaluation of the effectiveness and applicability of RDTs in the diagnosis of malaria.

## Background

Yunnan was one of the only two provinces in China where malaria was indigenous endemic cases as of 2013 [1, 2]. The number of malaria cases diagnosed by parasitology method (including microscopy and genetic test) in Yunnan Province took over 17.3% (533/3078) of China, and 45.0% (9/20) of highly endemic counties in 2014 [2]. Although the number of reported malaria cases in the Yunnan Province has been the highest in China since 2016, but all the cases were imported [3]. Among them, Myanmar (Burma) was the largest source of imported malaria cases, taking over 84.9% (477/562), while falciparum malaria was the main type of imported malaria from Africa (92.9%, 79/85). The patients were mainly farmers and businessmen who work abroad. Cases were found mainly in Tengchong, Ruili and Yinjiang counties along the China-Myanmar border. Therefore, Yunnan Province pays special attention to timely diagnosis and discovery of malaria spreading sources with measures such as vector control for stop malaria spreading and sensitivity monitoring for anti-malaria drugs [4–7]. A specific strategy, focused on the primary medical health institutions and individual clinics across the province, is to promote the use of immune rapid diagnostic tests (RDTs), such as Wonfo rapid diagnostic cassette which sensitivity was almost 95% for *Plasmodium falciparum* in 2008 [8]. The use of RDTs are up to ten thousands cassettes per year in order to ensure that the malaria cases in the far rural of Yunnan Province get timely screening, but malaria diagnosis must be confirmed pass through various stages of county-level, prefecture-level, and province-level malaria diagnostic laboratory. The confirmation methods include further parasitological microscopy by professionals in combination with genetic testing, in order to reach an accurate diagnosis of malaria and a general quality evaluation of RDTs diagnosis [6, 7, 9]. After several years of observation in Yunnan Province, false negative and false positive malaria cases by RDTs diagnosis occurred frequently, compared with the gold standard microscopic examination method. A systematic evaluation of the accuracy of RDTs diagnosis of malaria in Yunnan Province showed that nearly half of the RDTs were insensitive to about 3–33% of *P. falciparum* infections despite the tendency to overestimate the sensitivity and specificity [10]. The World Health Organization (WHO), after six rounds of RDT quality assessments, pointed out that the *P. falciparum* histidine-rich protein 2 (*Pf*HRP2) gene deletion may be the mainly biological factor accounting for failed diagnoses of falciparum malaria. Meanwhile, the WHO appeal to the regions with falciparum malaria epidemic worldwide for promptly clarification and validation of the *Pf*HRP2 (genes) deletion as well as its influence on the accuracy of RDTs diagnosis of malaria [11].

The target proteins of malaria RDTs diagnosis mainly include *Pf*HRP2, *P. falciparum* lactate dehydrogenase (pLDH) and *P. falciparum* aldolase (ALD). The first two are the detection targets of most malaria RDT products [10, 12], and *Pf*HRP2 is a specific diagnosis target protein for falciparum malaria. Unfortunately, since the existence of *P. falciparum* strains with *pfhrp2* gene deletion was first reported in the 1980s [13–15], the phenomena of *pfhrp2* gene deletion has been found in Peru [16], Mali [17], India [18, 19], Philippines [20], Senegal [21], Brazil [22], Yemen [23], Honduras [24]. Amoah et al. found that in Ghana the false negative rate by RDTs diagnosis for falciparum malaria was 52%, and the proportion of undetected *pfhrp2* gene was as high as 40%. In contrast, in areas with a false negative rate of 2%, only 22% of *pfhrp2* genes were undetected [25]; hence, Amoah et al. speculated that the spread of *P. falciparum* without the *pfhrp2* gene had increased the incidence of errors in RDT diagnosis of falciparum malaria in Ghana. The studies conducted by Akinyi et al. and Wurtz et al. indicated these phenomena of a combination of RDT diagnosis failures for falciparum malaria, and the *pfhrp2* gene entire deletion in *P. falciparum* isolates from Peru and Senegal [16, 26]. In Yemen [23], Angola [21], and India [19], entire deletion rates of the *pfhrp2* gene exon2 regions reached 9.5%, 10%, and 23%, respectively, indicating that these countries may have a higher risk of misdiagnosis with the *pfhrp2* (gene)-deficient strains when using *Pf*HRP2-target protein RDTs for falciparum malaria diagnosis. In 2014, Yang et al. detected the polymorphisms of *pfhrp2* gene in 20 *P. falciparum* isolates collected from the China-Myanmar border [27]. In 2015, Dong et al. also found the possibility of partial and entire polymorphisms of *pfhrp2* gene deletion in falciparum malaria case isolates from Yunnan Province [28]. Certainly, there are exceptions across the world, for example, Kenya has not yet found any entire deletion of *pfhrp2* gene in *P. falciparum* [29, 30]. However, given that Yunnan Province has not explored the reasons for the failure of RDT diagnoses of falciparum malaria, the polymorphism associated with *pfhrp2* gene deletion and its changes need to be further verified. More importantly, in response to the WHO’s appeal for the selection and improvement of RDTs, it is essential to understand the prevalence of *pfhrp2* gene deficiency, *Pf*HRP2 protein deficiency, the extent of antigenic variation, and to provide evidence for Yunnan Province to improve the quality of falciparum malaria RDT diagnoses [11, 30]. This study aimed to systematically analyse the data from an ongoing molecular epidemiological investigation on *pfhrp2* gene deletion in Yunnan Province.

## Methods

### Ethics statement

The study was approved by Yunnan Institute of Parasitic Diseases and by the Ethical Committee. Genetic testing experiment, etc. were performed on stored blood samples obtained as part of routine diagnostic work-up from patients with fever suspected of malaria. Although the absence of risk and the anonymous data processing are ensured among the study, consent from potential malaria patients need to be obtained during collecting blood samples.

### Study subjects and blood samples

Blood samples from falciparum malaria cases were continuously collected diagnosed and reported by Yunnan Province, and officially registered and approved by the “China Information System for Disease Control Prevention from January 2013 to December 2018. *P. falciparum* infection was parasitological confirmed by both microscopic examination [31] and genetic testing [32]. The filter paper blood samples were simultaneously used for polymorphic analysis of *pfhrp2* gene sources of falciparum malaria infection were confirmed by epidemiological investigation.

### *Plasmodium* DNA extraction and its normality verification

Three filter paper blood drops, each with a diameter of 5 mm, was taken, and *Plasmodium* genomic DNA was extracted according to the instructions of QIAgen Mini Kit (DNA Mini Kit, QIAamp company, Germany), and extracted genomic DNA liquid stored at − 20 °C for later use. The *pfcrt* gene exon2 region was amplified to verify whether the DNA extraction of the *Plasmodium* genome was normal [7].

### Amplification of the exon2 region of *pfhrp2* gene by nested PCR

The GenBank (https://www.ncbi.nlm.nih.gov/gene/) reference sequence (ID: nc_004329.3) was used to design primers and reaction conditions for PCR amplification of *pfhrp2* gene exon2 [18, 20, 33]. The forward and reverse primers for the first round of PCR amplification were 5′-TATCCGCTGCCGTTTT-3′ and 5′-CGCTATCCCATAAATTACAAAA-3′, respectively, and the primers for the second round were 5′-TGTGTAGCAAAAATGCAAAAGG-3′ and 5′-TTAATGGCGTAGGCAATGTG-3′, respectively, that amplified 1,374,236–1,375,059 bp in chromosome 8 (GenBank accession number, NC_004329.3), which coding region was from 57 to 301 aa of *pfhrp2* gene exon2 region, and the product size was expected to be about 824 bp. Both first and second PCR reactions contained 2.6 µL DNA template, 14 µL of 2× Taq PCR mixed system (containing Taq enzyme that was purchased from Qiagen Biotech (Shanghai), 0.7 µL each forward and reverse primers (20 µmol/L), and ddH_2_O for a volume of 25 µL. Both the conditions of first and second PCR reactions were as follows:95 °C for 5 min; 35 cycles of 95 °C for 30 s, 55 °C for 40 s, and 72 °C for 90 s; and 72 °C for 7 min. The amplified product of the second PCR was detected by 1.5% agarose, observed via 1.5% agarose gel electrophoresis (agarose and DNA standards were procured from Takara Biomedical Technology (Dalian). Then the positive products were sent to Shanghai Meiji Biomedical Technology Co., Ltd. for sequencing using the dideoxy chain-termination method. Sequenced data were analysed using Chromas Pro 2.33 software.

### Analysis of *pfhrp2* gene polymorphism and amino acid repeat sequence

DNA sequences from PCR product sequencing were aligned with the reference sequence of *pfhrp* gene exon2 region (GenBank accession number, PF3D7_08318000) using BLAST module of NCBI after splicing or transformation in the DNA Star Lasergene 7.1 software [33–36]. The sorted DNA sequences were converted to amino acid sequences using MEGA 5.04 software [33–36]. The DNA sequence of the *pfhrp2* gene exon2 region was analysed by Arlequin 3.5.2.2 software, and the haplotypes, the nucleic acid diversity index (PI) and expected heterozygosity (He) were calculated [33, 36]. Jalview 2.10.5 was used to prepare the Principal Component Analysis scatter diagram of the haplotypes. The amino acid repeat types of the *Pf*HRP2 peptide chain were counted, and the identification of the amino acid repeat types followed the principle of “long rather than short”. Such as, the “ AHHAHHAAD “ amino acid sequence was identified only as a type 2 repeat, not as a linkage between type 4 (AHH) and type 7 (AHHAAD), and the “AHHAHHATD” amino acid sequence was identified only as a type 14 repeat, not as a linkage between type 4 (AHH) and type 6 (AHHATD) [16–18, 20, 30]. *F* test was performed on the amino acid residues and sequence repeats in *pfhrp2* gene exon2 region using the SPSS16.0 software, and the inspection level was 0.05.

### Prediction of RDTs testing sensitivity via the *Pf*HRP2 peptide chain

The product of the number of *Pf*HRP2 repeat types 2 and 7 (type 2 × type 7) was used to distinguish the sensitivity of *Pf*HRP2 peptide chain reaction to RDTs. Assay products with sensitivity responses > 100, 50–100, 43, 44–49 and ˂43 to the RDTs were classified as very sensitive (group A) and sensitive (group B), borderline (group I) and non-sensitive (group C) peptides, respectively [30]. The response of RDTs for the *Pf*HRP2 peptide chain in geographical populations of *P. falciparum* was evaluated via the *T* test using SPSS16.0 software, and the inspection level was 0.05. Arc Gis10.1 was used to map the distribution of samples including normal sequencing *pfhrp2* gene exon2 and failed cases [36].

## Results

### Sample source and nested PCR amplification

A total of 306 blood samples were collected from falciparum malaria cases, 84.9% (259/306) from which the target *pfhrp2* gene exon2 region was amplified, with a fragment length of about 345 (not shown in electrophoresis)–927 bp. 47 blood samples was not amplified to cover the target *pfhrp2* gene exon2 region because its *pfcrt* gene exon2 fragment (150 bp) was amplified and identified as normal DNA extracted from the *Plasmodium* genome.

**Fig. 1:**
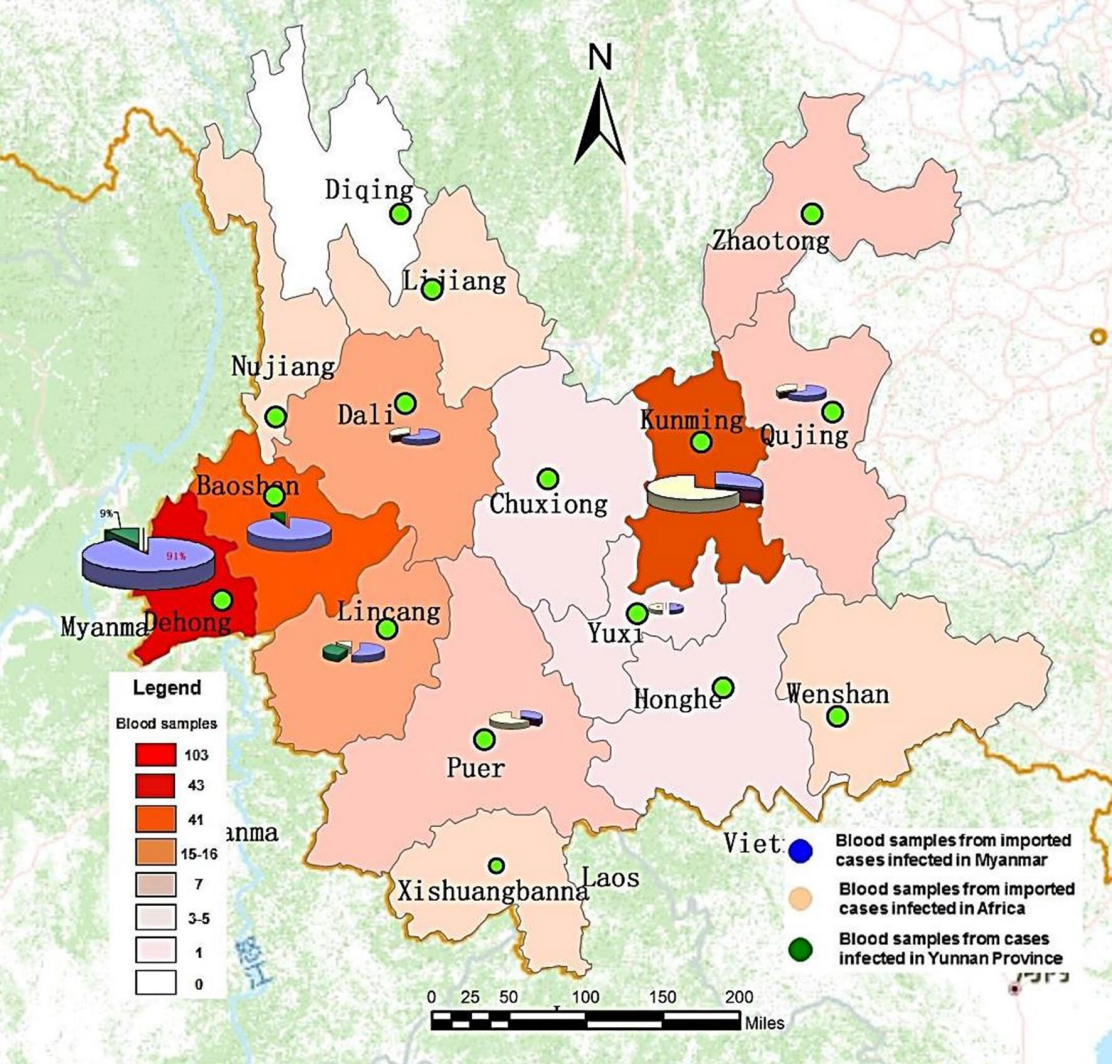
Distribution of falciparum cases blood samples including successful and unsuccessful PCR amplification of *pfhrp2* gene exon2 region. (1) The different colours from dark red to light red in administrative areas represented samples with *hrp2* gene. PCR amplication product that were collected from falciparum malaria cases found during study period, including Dehong, Kuming, Baoshan, Lincang, Dali, Pu’er, Qujing, Zhaotong, Nujiang, Lijiang, Xishuangbanna, Weishan, Honghe, Yuxi and Chuxiong –15 prefectures in Yunnan Province; White range indicates no falciparum malaria cases found during study period in this prefecture (only one) which is Diqing in Yunnan Province. (2) Pie charts represented failed amplification of samples with *hrp2* gene PCR. They were found in Dehong, Baoshan, Kuming, Dali, Lincang, Pu’er and Qujing 7 prefectures in Yunnan Province

Two hundred and fifty-nine blood samples has been through the PCR amplification products of *phhrp2* gene exon2 region, collected from prefecture falciparum malaria cases including Dehong (103 cases), Kunming (43 cases), Baoshan (41 cases), Dali (16 cases), Lincang (15 cases), Qujing (7 cases), Zhaotong (7 cases), Puer (7 cases), Wenshan (5 cases), Nujiang (4 cases), Xishuangbanna (4 cases), Lijiang (3 cases), Honghe (2 cases), Yuxi (1 case), Chuxiong (1 cases) (Fig. 1).

The cases information above collected 259 blood samples was shown in Table 1. The proportions of cases imported from Myanmar infection, imported from African countries infection and Yunnan indigenous infection were 59.5% (154/259), 36.3% (94/259) and 4.2% (11/259), respectively.

**Table 1:** Information of 259 falciparum malaria cases with their blood samples successful amplified out *pfhrp2* gene exon2 fragments

Years information	2013	2014	2015	2016	2017	2018	Total
Number	62	50	68	26	25	28	259
1. Age^a^							
Maximum	58	56	59	58	57	49	56
Minimum	8	2	10	15	8	1	7
Average	33	31	33	38	31	33	33
2. Gender^b^							
Male	56	42	67	26	22	26	239
Female	6	8	1	0	3	2	20
3. Infection source^c^							
Myanmar	36	40	49	14	8	7	154
Africa	21	4	19	12	17	21	94
Yunnan indigenous	5	6	0	0	0	0	11

^a^Years old; ^b^Numbers; ^c^Identified by epidemiological investigation

Forty-seven blood samples which the *pfhrp2* exon2 region was not amplified were collected from 8 prefectures falciparum malaria cases in Yunnan province, including Dehong, Baoshan, Kunming, Lincang, Dali, Puerr, Yuxi, and Qujing. The proportions of these cases imported from Myanmar infection, imported from African countries infection and Yunnan indigenous infection were 63.7% (29/47), 23.4% (11/47) and 14.9% (7/47), respectively. Yunnan indigenous infection cases blood samples with failed amplification of *pfhrp2* exon2 region were only limited to three prefectures including Dehong, Baoshan and Lincang. On the contrary, the blood samples with failed amplification of that gene fragment which were collected from Kunming and Qujing prefectures involved a large number of cases imported from Africa counties infection (Fig. 1).

### Polymorphism of DNA sequences

A total of 250 DNA sequences of the *pfhrp2* exon2 region were obtained by sequencing 259 PCR amplification products, with lengths ranging from 345 to 927 bp. Polymorphic analyses showed that there were 151 haplotypes, Hap_001–Hap_151, and that PI and He were 0.169 and 0.983, respectively. There were 87, 77 and 10 haplotypes in 146 Myanmar geographical populations, 89 African geographical populations and 15 Yunnan geographical populations which were traced by epidemiological investigation, respectively. The PI and He of the three geographical populations were 0.167 and 0.974, 0.219 and 0.997, 0.249 and 0.981, respectively.

**Fig. 2:**
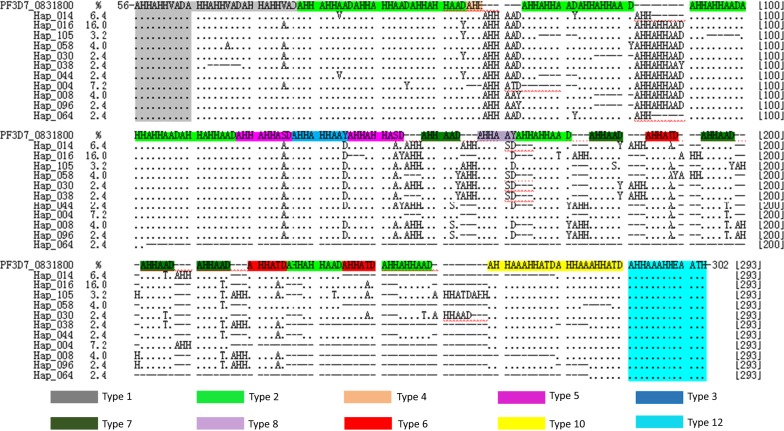
Alignment mapping of amino acid chains from different haplotypes. The alignment reference sequence was PF3D7_0831800. The alignment region of amino acid chain was from 57th to 301th aa in *pfhrp2* gene exon2 region

Hap_016, with a DNA sequence length of 771 bp, was present in the largest proportion of the 151 haplotypes, accounting for 16.0% (40/250). The remaining haplotypes were Hap_004 (7.2%, 18/250), Hap_014 (6.4%, 16/250), Hap_008 and Hap_058 (4.0%, 10/250), Hap_105 (3.2%, 8/250), and Hap_030 (2.4%, 6/250) (Fig. 2). Seventeen haplotypes including Hap_005, twelve haplotypes including Hap_017, and twenty-eight haplotypes including Hap_026, accounted for 1.6% (4/250), 1.2% (3/250), and 0.8% (2/250), respectively. The others’ eighty-three haplotypes with only one sequence had a frequency of 0.4% (1/250).

PCA scatter plots of 151 haplotypes showed that the haplotypes with smallest score (− 12.910) (Y: − 14.041, Z: 3.889) and the largest score (13.543 (Y: − 4.160, Z: 34.599) in the X-axis direction were two sequences from Myanmar geographical isolates. The haplotypes with the smallest score (− 13.685) (X: 11.954, Z: 0.804) and the largest score (14.593) (X: 1.744, Z: − 2.442) in the Y-axis direction included one Myanmar isolate and one African isolate. Haplotypes with the smallest score (− 12.873) (X: 6.167, Y: − 8.421) and the largest score (34.599) (X: 13.543, Y: − 4.160) in the z-axis direction were also from two Myanmar isolates.

**Fig. 3:**
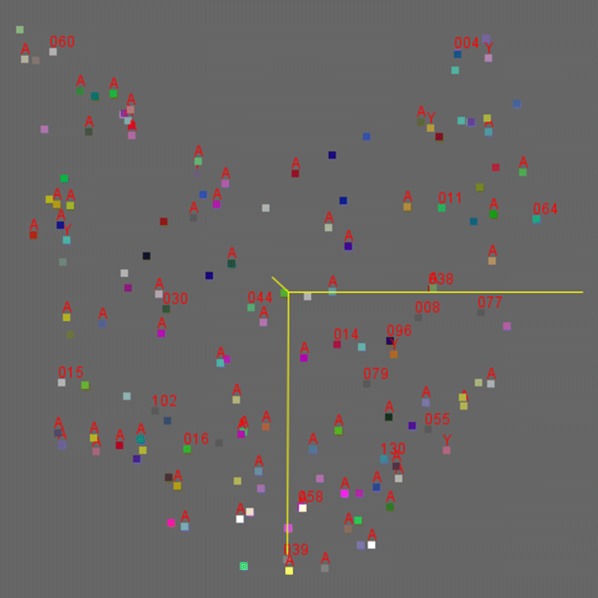
PCA scatter plots of DNA sequences haplotypes of *hrp2* exon2 region from different infection source *P. falciparum* isolates. (1) The horizontal yellow line was the X axis, and vertical yellow line the Y axis, and left inclined yellow line the Z axis. (2) The different color blocks represented haplotypes, in which yellow blocks were these haplotypes with the minimum PCA value and red blocks were those with the maximum PCA value. (3) The number labels on the block were the haplotype names with frequencies greater than 0.8%. A labels block are haplotypes with only sequence of African isolates, and Y labels block are haplotypes with only sequence of Yunnan Province local isolates, and no labels block are haplotypes with only sequence of Myanmar isolates

Eleven high frequency haplotypes, including “Hap_014″, “Hap_016″, “Hap_105”, “Hap_058”, “Hap_030”, “Hap_038”, “Hap_044”, “Hap_004”, “Hap_008”, “Hap_096”, and “Hap_064”, with the PCA locations, X: 4.134, Y: 2.598, Z: 1.951; X: − 3.927, Y: 8.212, Z: 2.775; X: − 6.644, Y:− 8.952, Z: − 2.350; X: 2.480, Y: 11.215, Z: − 3.569; X: − 4.944, Y: 0.798, Z: 0.178; X: 9.492, Y: − 0.388, Z: − 8.409; X: − 0.427, Y: 0.694,Z: − 0.463; X: 10.719, Y: − 12.883, Z: − 6.025; X: 8.430, Y: 1.204, Z: 2.151; X: 6.962, Y: 2.430, Z: 0.230; and X: 13.543, Y: − 4.160, Z: 34.599, respectively, were discrete and non-overlapping. Also, the haplotype which only composed of the sequences from Myanmar isolates, Africa isolates or Yunnan isolates, did not show any aggregation tendency (Fig. 3).

Following the translation of 250 DNA sequences into amino acid sequences, the longest was 309 aa of haplotype Hap_114, and the shortest was 115 aa of Hap_064 (Fig. 2), with an overall average length of 245 aa (± 26.256). Of these, the average amino acid chain lengths of Myanmar population, African population and Yunnan population were 242 (± 29.163) aa, 251 (± 19.750) aa and 241 (± 19.721) aa, respectively, and the difference in amino acid chain length among different geographical populations was statistically significant (*F *= 3.227, *P *= 0.041).

### Structural characteristics of *Pf*HRP2 peptide chains

A total of 19 amino acid repeat types were found in 250 amino acid sequences (Table 1). Of these, type 2 (AHHAHHAAD) and type 12 (AHHAAAHHEAATH) repeats occurred in all (100%, 250/250) the amino acid sequences, with type 12 repeats as the final sequence (Fig. 2). For the initial sequence repetitions of N-terminal, 98.0% (245/250) were type 1 (AHHAHHVAD) (Fig. 3), and 2.0% (5/250) were type 2 (AHHAHHAAD). When each amino acid sequence was used to count the repetition types, type 2 repetition repeated the most frequent (12.32 times/sequence), and others occurred as follows: 4.79 times/sequence of type 7, 2.99 times/sequence of type 6, 2.59 times/sequence of type 1, and 1.52 times/sequence of type 10 (Table 2).

**Table 2:** Amino acid repeat types and number of *pfhrp2* gene exon2 region in different geographical isolates from Yunnan Province

Repeat types	Characteristic repeat sequence	Number (%)	Mean	Myanmar			Africa			Yunnan indigenous		
				Mini	Maxi	Mean	Mini	Maxi	Mean	Mini	Maxi	Mean
1	AHHAHHVAD	245 (98.0)	2.59	1	5	2.39	1	6	2.35	1	4	2.3
2	AHHAHHAAD	250 (100.0)	12.32	1	19	2.37	7	16	2.31	11	16	13.6
	AHHAHHAAH	5 (2.0)	0.04	1	3	2.33	1	3	2.0	0	0	0
	AHHAHHAPD	14 (5.6)	0.18	1	3	2.0	1	9	3.80	0	0	0
	AHHAHHAPH	9 (3.6)	0.18	5	5	5.0	1	10	4.88	0	0	0
3	AHHAHHAAY	235 (94.0)	1.33	1	5	1.36	1	2	1.37	1	2	1.2
	AHHAHHAPY	5 (2.0)	0.02	1	1	1.0	1	1	1.0	0	0	0
4	AHH	116 (46.4)	1.05	1	34	2.37	1	3	1.6	1	2	1.67
5	AHHAHHASD	166 (66.4)	0.80	1	6	1.16	1	5	1.33	1	2	1.17
6	AHHATD	246 (98.4)	2.99	1	7	2.86	1	7	3.19	2	4	2.8
7	AHHAAD	246 (98.4)	4.79	1	11	4.45	1	11	4.35	1	6	3.9
	AHHAPD	15 (6.0)	0.12	1	3	1.22	2	4	3.17	0	0	0
	AHHAPH	6 (2.4)	0.06	1	3	2.0	2	3	2.75	0	0	0
8	AHHAAY	231 (92.4)	1.11	1	2	1.15	1	2	1.1	1	3	1.4
	AHHAPY	5 (2.0)	0.04	1	3	2.0	1	3	1.67	0	0	0
10	AHHAAAHHATD	228 (91.2)	1.52	1	4	1.63	1	3	1.78	1	2	1.67
12	AHHAAAHHEAATH	250 (100.0)	1.00	1	1	1	1	1	1	1	1	1
13	AHHASD	33 (13.2)	0.14	1	1	1	1	2	1.33	1	1	1
14	AHHAHHATD	20 (8.0)	0.08	1	1	1	1	1	1	1	1	1

The average repetition times for types 1, 2, 3, 4, 5, 6, 7, 8, 10, 13 in Myanmar geographical population, Africa geographical population and Yunnan geographical population were not statistically significant (*F *= 0.032, 1.296, 0.370, 0.186, 0.540, 1.130, 0.263, 2.282, 0.645, 3.125, respectively; *P *> 0.05). Moreover, the three amino acid repetitions of “AHHAHHAAH”, “AHHAHHAPD” and “AHHAHHAPH” similar to type 2, the “AHHAHHAPY” similar to type 3, the “AHHAPD” and “AHHAPH” similar to type 7, and the “AHHAPY” similar to type 8 only appeared in the sequences of Myanmar population and African population (Table 2).

### RDT testing sensitivity for *Pf*HRP2 peptide chains

The sensitivity testing of RDTs for *Pf*HRP2 peptide chains based on predictions of type 2 and type 7 amino acid repetition frequencies suggested that the products of the number of *Pf*HRP2 repeat types 2 and 7 (type 2 × type 7) were between 52 and 99 in group B accounted for most *Pf*HRP2 peptide chains (50.0%, 125/250). The shared advantages in Myanmar, Africa, and Yunnan was also reflected, but the separation plant taxa of group B peptides in Myanmar were lower than African and Yunnan isolates (*t *= 28.802, *P* < 0.05) (Table 3). The product between 100 and 182 had the least proportion of group A *Pf*HRP2 peptide chains (9.6%, 24/250), and the proportion of group A peptide chains isolated from Myanmar was significantly lower than those from African and Yunnan isolates (*t *= 12.306, P < 0.05) (Table 3). On the contrary, the proportion of group I and group C peptide chains from Myanmar isolates was significantly higher than those of African (*t *= 20.562, *P *< 0.05) and Yunnan (*t *= 38.045, *P *< 0.05) isolates (Table 3).

**Table 3:** Comparison of test sensitivity types of *Pf*HRP2 peptide chains from different infectious sources *P. falciparum* isolates in Yunnan Province

Groups	No. *Pf*HRP2 (%)	Mini of Type 2 × Type 7	Max of Type 2 × Type 7	Myanmar		Africa		Yunnan local		*T test (p value*^)^
				No. (%)	Means of Type 2 × Type 7 (*Sd*)	No.(%)	Means of Type 2 × Type 7 (*Sd*)	No.(%)	Means of Type 2 × Type 7 *(Sd*)	
A	24 (9.60)	100	182	11 (7.38)	117.09 (8.276)	11 (12.79)	123.55 (24.825)	2 (13.33)	105.0 (0.000)	12.306 (0.000)
B	125 (50.0)	52	99	64 (42.95)	75.20 (14.634)	52 (60.47)	75.79 (13.934)	9 (60.0)	80.67 (8.047)	28.802 (0.000)
I	33 (13.20)	44	48	22 (14.77)	45.50 (1.626)	9 (10.47)	45.0 (1.732)	2 (13.33)	48.0 (0.000)	13.143 (0.000)
C	68 (27.20)	6	42	52 (34.90)	25.75 (10,895)	14 (16.28)	29.21 (7.138)	2 (13.33)	20.50 (0.000)	20.562 (0.000)
Total	250	6	182	149	56.65 (30.189)	86	71.09 (30.456)	15	71.53 (26.643)	38.045 (0.000)

## Discussion

*pfhrp2* gene (1064 bp) is located in the chromosome 8 telomere area [35], and consists of one non-coding region, one intron area, and one histidine and alanine tandem repeat as dominant in coding regions. The alanine and histidine tandem repeats about 1000 bp, and the secretory area at upstream of the coding area 5’- terminal constitutes the exon2 region (a total of 305 coding amino acids). Due to the instability of the telomeres, the frequent recombination in *pfhrp2* gene region is prone to lead to increased mutations and a more complex diversity of amino acid tandem repeats [16, 37–40]. The deletion of *pfhrp2* gene exon2 [15] and the protein variation of expressed *Pf*HRP2 not only affect the accuracy of RDTs diagnosis falciparum malaria against *Pf*HRP2 target protein, but also lead to the potential harm of changing the habits of *Plasmodium* and even changing the prevalence intensity of malaria [41].

*Pf*HRP2 is not only involved in transforming the toxicity for pigment to poison *Plasmodium* parasites, but also plays a role in reshaping the structure of the erythrocyte membrane to enhance the immune escape of malaria parasites [42–44].

In this study, the polymorphism of *pfhrp2* gene in the 57–301 aa coding region, which is also a dense repeat region of alanine and histidine in the exon2 region, of *P. falciparum* isolates in Yunnan province in the past 6 years was analyzed. Of the 306 blood samples, 250 samples had obtained the target DNA sequence (345–927 bp), which are similar in length from the geographical isolates in Senegal [21], Nigeria [23], Yemen [29], and Mozambique [45, 46]. There were 151 haplotypes in the DNA sequence, with a PI of 0.169, which is higher than those in Mali (0.005) and Angola (0.011) [21], and lower than that in Eritrea (0.580) [45]. Furthermore, the haplotype which there was only one DNA sequence accounted for 33.2% (83/250) of the 250 DNA sequences, which was less than was observed by Deme et al. (43.3%, 29/67) [21] and Baker et al. (56.6%, 259/458) [29] in their analyses of mixed samples in Africa. This result suggests that the differentiation of *pfhrp2* gene in the sample isolates was greater than that previously reported in African isolates [21, 29], which may be due to the inclusion of complex geographical populations and highly differentiated isolates in the sample. The nucleic acid diversity index (PI) of *pfhrp2* gene from the Yunnan isolates was as high as 0.249, which is the highest among Myanmar, African and Yunnan groups.

The 250 *Pf*HRP2 peptide chains derived from the DNA sequence were between 115 and 309 aa in length, with an average of 245 aa, from total cases isolates. The length of 242 aa from the Myanmar isolates was consistent with the study conducted by Baker et al. [29], but shorter than Haiti’s 262 aa, Brazil’s 261 aa and Vietnam’s 274 aa. This result further enriched the polymorphism of the *pfhrp2* gene exon2 region in Southeast Asia. It is interesting to note that for the same infection across the same period, when we compared the *pfhrp2* gene exon2 area and DNA sequences, and found that the peptides and amino acids of *pfhrp2* with the same chain length as other infection isolates have different sequence structures. A prompt investigation of *pfhrp2* gene mutation and gene exon2 region diversity may have potential gene traceability between different *P. falciparum* isolates.

Regarding the *Pf*HRP2 peptide amino acid repeat feature recognition, all 250 peptide chains showed 19 kinds of repeats, including type 1, type 2 and its three variation types, type 3 and its one variation type, type 4, type 5, type 6, type 7 and its two variation types, type 8 and its one variation type 10, type 12 and type 13 (Table 1). The repeats of types 9 and type 11 was not observed in this study. These repetitive features differed from those 13 types obtained from India isolates by Kumar et al. [18, 47], 12 repeating characteristics from Senegal isolates by Deme et al. [21], 14 repeating patterns from mixed isolates collecting from many countries by Baker et al. [29], and 39 repeating patterns from Kenya isolates by Nderu et al. [30]. However, in all these studies, amino acid residues repetition of types 1–8, type 10 and type 12 were found. The pattern starting with type 1 repetition and ending with type 12 repetitions were relatively fixed, and they were found in all peptide chains. However, further comparison with different studies showed that the identified amino acid repeat types of the *Pf*HRP2 peptide chain must be in accordance with the stable identification criteria, especially when the amino acid repeat types in *Pf*HRP2 exon2 region were used for predicting RDT reactivity for the *Pf*HRP2 peptide chain. Although the amino acid repeat sequence of *Pf*HRP2 peptide chain were summarized as type 1, type 2, type 3, type 4, type 5, type 6, type 7, type 8, type 10, type 12, type 13, type 14 and other variants [18, 20, 30], the recognition and counting of repeat types can only be done manually. Therefore, potential counting differences might exist caused by different counting strategies. For example, type 2 (AHHAHHAAD) was counted as a linkage between type 4 (AHH) and type 7 (AHHAAD), while type 14 (AHHAHHATD) was counted as a linkage between type 4 (AHH) and type 6 (AHHATD), which resulted in trading off and taking turns of type 2, type 4, type 6, and type 7. Due to the complex recognition principle of “long rather than short” in this study, the differences may occur in the recognition and counting of type 2, type 7 and type 4 repetitions compared with those of Yang et al. [27] and Li et al. [48]. For the detection of blood samples from falciparum malaria cases in Yunnan province, the average number of repetitions of type 7 and type 4 in this study were 4.8 times and 1.0 time, respectively, while Yang’s type 7 was 15.6 times, with no type 4 repeat characteristics [27], showing a significant difference.

Studies have demonstrated that antigen variation is a risk factor for the unstable quality of falciparum malaria diagnosed by RDTs based on immune response [26, 49], and the epitope region of the *Pf*HRP2 peptide chain is thought to be mainly confined to its central region [50]. Therefore, rapid prediction of RDTs testing sensitivity for *Pf*HRP2 peptide chain by type 2 repeat times and type 7 repeat times have become a trend [18, 20, 26, 29, 30]. Studies conducted by Kumar et al. [18] and Wurtz et al. [26] found that when the products of type 2 and type 7 repeat times are < 43 (group C chain), RDTs will find it difficult to detect the presence of this *Pf*HRP2 chain. Fortunately, the *Pf*HRP2 peptide chain belonging to group C was not the dominant species in the current study samples. In this study, the *Pf*HRP2 peptide chain of group C only accounted for 27.3% (68/250), but it was still higher than that of the samples from Senegal (7.4%, 9/122) [26] and in Kenya (9.8%, 24/244) [30]. In contrast, the dominant species were *Pf*HRP2 peptide chains belonging to group B, accounting for 50.0% (125/250), which was lower than 71.3% (87/122) [26] and 75.8% (185/244) [30] obtained from previous studies. This suggested that there may be more RDT detection failures than those for African isolate alone in this study, which was mixed with many Myanmar isolates (59.6%, 149/250). Unfortunately, matched pair study between falciparum malaria diagnosed using RDT in Yunnan province and the polymorphic structure of *Pf*HRP2 peptide chain have not been implemented. Therefore, it is not possible to infer whether *Pf*HRP2 peptide chain variation affects falciparum malaria diagnosis in Yunnan province.

In this study, *pfhrp2* gene was not amplified in 15.36% (47/306) of the samples when extracting *P. falciparum* genomic DNA was confirmed to be normal from these samples. Still, the author could not ensure that the *P. falciparum* isolates in these samples can be considered entire deficiency of *pfhrp2* gene. First, *pfhrp2* gene deletion was not verified through Cheng’s method [51]. Second, although the nested PCR method was used in this study to amplify *pfhrp2* gene with higher amplification efficiency than Kumar et al. [18] and Baker et al. [20], the amplified fragment only covered the region from the intron to 248 bp downstream of *pfhrp2* gene, and the sensitivity to confirm *pfhrp2* (gene) deletion may not have been high enough. Parr et al. [52] and Trouvay et al. [53] adopted whole genome sequencing, while Gupta et al. [46] and Ranadive et al. [54] amplified the region to form the upstream and downstream of *pfhrp2* gene extension [51], while the phenomenon of *pfhrp2* gene deletion was not found or rarely found. However, more phenomenon of *pfhrp2* gene deletion were found only in amplifying exon2 region alone [20, 36, 55], which suggests that analysis of the exon2 region of *pfhrp2* gene alone may be less sensitive than whole-genome or upstream and downstream extension assays for confirmation of *pfhrp2* gene entire deletion. Third, the failure to amplify the *pfhrp2* gene above 47 samples does not rule out the effect of low *Plasmodium* density. Gupta et al. indicated that the analysis on *pfhrp2* gene deletion was unreliable when *Plasmodium* density is only 3 ~ 330 parasites/µl. Therefore, the study conducted by Beshir et al. excluded samples with *Plasmodium* densities < 5 parasites/µl during validation of *pfhrp2* gene deletion. In conclusion, the certification of *pfhrp2* gene entire deletion must be rigorous, as the WHO requires that programme must adjust their RDTs product when the proportion of *pfhrp2* gene entire deletion of *P. falciparum* achieve to 5% in a population within an area [56].

This study comprehensively analysed the *pfhrp2* gene polymorphism from falciparum malaria cases isolates from January 2013 to December 2018 in Yunnan province. The current *pfhrp2* gene polymorphism situation from falciparum malaria case isolates in Yunnan province was reflected due to good continuity and systemic characteristics of these samples. The results of the study will be the basis for further verification of *pfhrp2* gene entire deletion and screening of optimal epitopes of *Pf*HRP2 peptide chain. However, this study include several limitations: (1) due to the inability to determine the *Plasmodium* density in some blood samples, the effect of low protozoan density on the confirmation of *pfhrp2* gene deletion cannot be ruled out; (2) polymorphic and deletion of *pfhrp3* gene, which are considered as early signs of *pfhrp2* gene deletion [15], were not conducted due to limited human resources; and (3) whether the *Pf*HRP2 peptide chains exists or not in blood samples was not examined in parallel with RDT products in the study.

The study conducted by Watson et al. indicated that low prevalence of malaria and the high frequency for treatment could increase the risk for inappropriate use of antimalarial drugs because malaria might be misdiagnosed by RDTs, which will also lead to transitionally screening on *pfhrp2*-deficient plasmodium [57]. Currently, there was very low prevalence of malaria and high frequency of malaria-associated medical treatment in China. However, China was not a part of the WHO’s global RDT quality monitoring network, and the systematic quality tracking of RDT products were not conducted. Therefore, whether the use of RDTs will accelerate screening for *pfhrp2*-deficient *Plasmodium* deserves attention. Further rapid verification of the entire deletion of *pfhrp2* gene from *P. falciparum* in China deserve higher priority. Fortunately, a more sensitive and specific sequencing analysis method for *pfhrp2* gene deletion has developed.

## Conclusion

In conclusion, this study firstly, provides the *pfhrp2* gene diversity information of isolates from falciparum malaria cases diagnosed and reported by Yunnan Province, China. Although the presence of *pfhrp2* gene entire deletion is uncertain, the results are a general reflection of the presence of *pfhrp2* gene polymorphism in Yunnan Province since the study samples are from official cases reported over the years. Therefore, it is necessary that the effectiveness and applicability of RDTs in diagnosis falciparum malaria in Yunnan province should be evaluated in time and it is important that the *pfhrp2* gene-deficient *P. falciparum* isolates should not be excessively screened by blindly extending the use of RDTs.

## Data Availability

Not applicable.
